# Use of point-of-care tests in pharyngotonsillitis – a registry-based study in primary health care

**DOI:** 10.1080/02813432.2024.2416671

**Published:** 2024-10-25

**Authors:** Jon Pallon, Katarina Hedin

**Affiliations:** a Department of Clinical Sciences in Malmö, Family Medicine, Lund University, Malmö, Sweden; b Department of Research and Development, Region Kronoberg, Växjö, Sweden; c Futurum, Region Jönköping County, Sweden; d Department of Health, Medicine and Caring Sciences, Linköping University, Linköping, Sweden

**Keywords:** Pharyngotonsillitis, rapid antigen detection test, C-reactive protein, point-of-care tests, primary health care, antibiotic prescribing

## Abstract

**Background:**

Point-of-care (POC) tests, including C-reactive protein (CRP) tests and rapid antigen detection tests (RADT) for group A streptococci (GAS), are widely used in Swedish primary health care (PHC). This study quantifies their use in pharyngotonsillitis and explore their association with antibiotic prescribing.

**Material and methods:**

Retrospective data from 2012–2016 in Region Kronoberg, Sweden, included all PHC visits with a pharyngotonsillitis diagnosis. Patient characteristics, test usage and antibiotic prescriptions were linked by visit date and personal identification number. Descriptive statistics were used for POC test analysis. Logistic regression assessed the association between CRP levels and antibiotic prescribing.

**Results:**

Of 24,237 visits, 68% included RADT and 36% included a CRP test, with 89% of CRP tests performed alongside RADT. CRP testing was more frequent in patients with negative (56%) than positive RADTs (42%) (*p* < .001). Overall, 66% of RADTs were positive. Median CRP levels were 23 mg/l for positive RADT and 31 mg/l for negative RADT (*p* < .001). Antibiotics were prescribed for 95% of positive RADTs and 43% of negative RADTs (*p* < .001). In patients with negative RADTs, CRP testing was associated with higher antibiotic prescribing (57%) compared to no CRP testing (26%) (*p* < .001). Among these patients, CRP levels were associated with prescribing (aOR 1.032; 95% CI 1.029–1.035; *p* < .001), with 50% of prescriptions occuring at CRP levels ≤ 20 mg/l.

**Conclusion:**

The use of RADTs and the proportion of positive test were higher than expected, indicating inappropriate use and diagnostic bias. CRP testing, contrary to guidelines, was common and associated with increased antibiotic prescribing.

## Background

Pharyngotonsillitis – i.e. acute sore throat – is one of the most common infections in primary health care (PHC) and accounts for a large proportion of anti­biotic prescriptions [1,2]. Both viruses and bacteria can cause pharyngotonsillitis [3], but most guidelines worldwide focus on diagnosing *Streptococcus pyogenes* (group A streptococci; GAS), which is considered the most important pathogen [4]. In addition to GAS, *Streptococcus dysgalactiae* subspecies *equisimilis*, also known as group C and G streptococci [5,6] and *Fusobacterium necrophorum* have been suggested as important pathogens [7,8].

As the clinical presentation overlaps greatly between different pathogens, several clinical decision rules have been developed to help identify GAS and direct antibiotic treatment to these patients [9]. One of the most widely used decision rules is the Centor score, which gives one point each for fever, lymphadenitis, tonsillar coating and absence of a cough and has a positive predictive value for GAS of 56% given the maximum of four points [10]. To further heighten the diagnostic accuracy, many guidelines recommend the use of rapid antigen detection tests (RADT) for GAS [4], which have a sensitivity and specificity of >85% [11]. These tests are specific for GAS and will not detect other streptococci or bacteria. However, the RADT cannot distinguish between a true GAS infection and a viral infection with concomitant GAS carriage. In Sweden, the national guideline advocates the use of RADT for patients with a Centor score of 3–4 and a presumed benefit from antibiotic treatment, emphasising antibiotic treatment solely for GAS-positive patients. Furthermore, it recommends that patients with typical viral symptoms, such as cough and runny nose, should not be tested or treated [12].

In PHC in Scandinavia, point-of-care (POC) tests for C-reactive protein (CRP) are widely used for various infections [13–16]. Although the inappropriate use of CRP tests can lead to increased antibiotic prescribing [15,17], a recent meta-analysis suggested a reduction in antibiotic prescribing for acute respiratory infections with CRP testing [18]. Despite this, evidence supporting CRP testing for pharyngotonsillitis is weak [19] and no guideline recommends its use [4]. In fact, the current Swedish guideline from 2012 explicitly advises against CRP testing [12]. In Denmark, nevertheless, CRP is commonly used in conjunction with a RADT for patients with pharyngotonsillitis [14,16]. In Sweden, two studies from 2004 found that a CRP test was used in 17–19% of patients with pharyngotonsillitis [13,17]. However, there is a lack of comprehensive observational data from the period after the introduction of the current guideline, except for a small prospective study conducted between 2012 and 2014, which reported a 50% use of CRP [20]. Additionally, it remains unclear whether CRP levels correlate with antibiotic prescribing in pharyngotonsillitis, although studies on respiratory infections, including acute sore throat, suggest this association [14,15].

This study aims to use pre-pandemic registry data (2012–2016) to quantify RADT and CRP test usage in patients diagnosed with pharyngotonsillitis in Region Kronoberg, Sweden and to explore the association between test results and antibiotic prescribing. We hypothesise that CRP – contrary to guidelines – is often used in patients with pharyngotonsillitis, and that CRP level is associated with antibiotic prescribing in patients with a negative RADT.

## Material and methods

This study is based on a new analysis of registry data collected between 2012 and 2016 previously extracted to study the association between bacterial aetiology, antibiotic prescribing and clinical course in patients diagnosed with pharyngotonsillitis in PHC [21]. The paper is reported in accordance with the RECORD statement [22].

### Study population and setting

This study was conducted in Region Kronoberg, a county located in southern Sweden that had a median population of 189,292 during the study period. Swedish health care is tax-funded and equally accessible to all inhabitants as part of the welfare system. The welfare system is based on about 1200 PHC centres (PHCC) located throughout the country as the first line of contact with the possibility to referral to hospital if necessary. The absolute majority of respiratory infections are primarily assessed in a PHCC. Of the 34 PHCCs in Region Kronoberg during the study period, 31 participated in the study. In addition, two out-of-hours centres (open between 17:00 and 21:00) participated in the study. In Region Kronoberg, patients who contact their PHCC are first assessed over the telephone by a triage nurse, who decides if a physician’s visit is necessary. Every visit to a physician requires a diagnosis code according to the 10th revision of the International Statistical Classification of Diseases and Related Health Problems (ICD-10) or its modified Swedish PHC edition (KSH97-P) [23].

### Data extraction

Data on pharyngotonsillitis for 2012–2016 were extracted retrospectively from the comprehensive electronic medical record (EMR) system (Cambio Cosmic, Cambio Healthcare Systems, Linköping, Sweden), which covers both PHC and hospitals in Region Kronoberg. Visit identification number, date, time and Swedish personal identification number were used for linkage. For this study patient data regarding age, sex, RADTs, POC CRP tests and antibiotic prescriptions were used.

In the first step, all visits to PHC with a diagnosis code for pharyngotonsillitis (J02x or J03x) were selected. Codes that only indicated sore throat as a symptom, such as R07 (pain in throat), were omitted, as were codes for other upper respiratory infections, such as J06 (unspecified site). In the second step, six exclusion criteria were applied: (1) a diagnosed pharyngotonsillitis the last 30 days; (2) a diagnosed complication (defined as peritonsillitis, media otitis, sinusitis, lymphadenitis or sepsis) the last 30 days; (3) incomplete data of the last 30 days (i.e. the first 30 days of the study); (4) antibiotic prescription the last 30 days; (5) a complication diagnosed on the same day as the visit and (6) prescription of an antibiotic not indicated for a sore throat. Phenoxymethylpenicillin (penicillin V), cefadroxil and clindamycin are recommended in the Swedish guideline [12]. Apart from the guideline, amoxicillin, erythromycin and azithromycin are also approved by the Swedish Medical Products Agency for treating pharyngotonsillitis. As RADTs and POC CRP tests are registered separately from visits and diagnosis codes in the EMR system, all data on these tests in Region Kronoberg during the study period were extracted.

### Point-of-care tests

The RADT kit for GAS used during the study period was QuickVue Dipstick Strep A (Quidel Corporation, San Diego, CA, USA), a lateral-flow immunoassay that detects both viable and nonviable organisms [24]. The CRP test used was Afinion^™^ CRP assay (Abbott Laboratories, Abbott Park, Illinois, USA), a rapid test for quantitative determination of CRP with a measuring range of 5–200 mg/l.

### Statistical methods

Data were cleaned and analysed using Excel 2019 (Microsoft, Redmond, WA, USA) and SPSS 27 software (IBM, Armonk, NY, USA). Categorical data were compared with two-sided Pearson Chi square test and with one sample Chi square goodness-of-fit test. *p* Values < .05 were considered significant. CRP was described as median (interquartile range [IQR]) due to non-normal distribution and a constricted measurement range (5–200 mg/l). In line with clinical practice, CRP level < 5 was regarded as zero. Mann-Whitney U test was used to compare median CRP levels and CRP distribution between two independent groups. The association between CRP level and the proportion of patients who were prescribed antibiotics were calculated partly for each CRP level and partly as a moving average of each CRP level ± 5 levels together with a 95% binomial proportion confidence interval (CI) for the moving average. To study the relationship between CRP level (exposure, expressed as an integer from 0–200) and antibiotic prescribing (binary outcome), logistic regression with 95% CI was used. A model was constructed to control for age (ratio variable) and gender (nominal) as possible confounders. Since the RADT outcome was thought to act as an effect modifier on CRP, patients were stratified with regard to RADT result (positive/negative) before analysis. Because the data was based on routine health data, and the data set was already extracted when planning the study, it was not possible to control for possible confounders such as clinical severity, individual signs and symptoms, duration of symptoms, medication, co-morbidity and immunosuppression.

## Results

**Figure 1.**
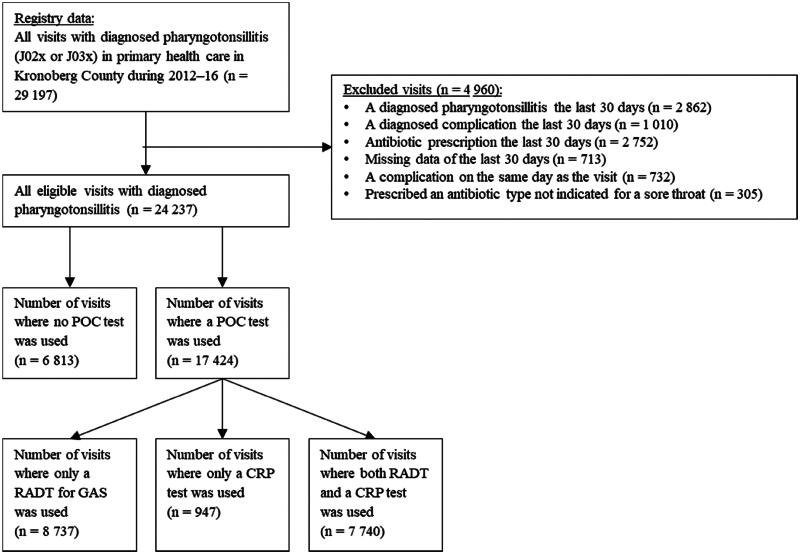
Flowchart of inclusion.

We identified 29,197 visits in PHC with diagnosed pharyngotonsillitis (J02x or J03x) during the study period 2012–2016. Of these, 4960 (17%) visits were excluded and 24,237 were considered eligible (Figure 1), resulting in 19,076 unique patients.

### Use of point-of-care tests

**Figure 2.**
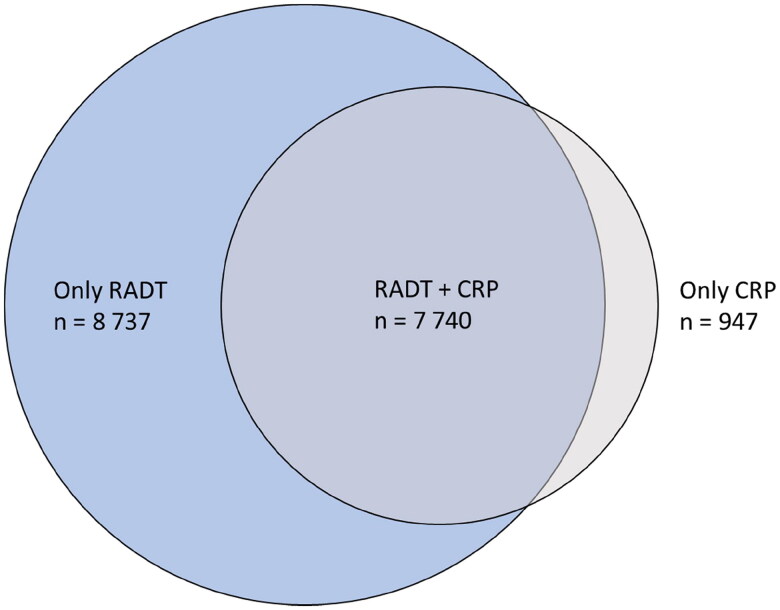
Venn diagram illustrating the use of point-of-care tests. In the 24,237 eligible visits with a diagnosed pharyngotonsillitis, a point-of-care test was used in 17,424 (72%) cases. RADT: rapid antigen detection test for group a streptococci; CRP: C-reactive protein test.

In the 24,237 eligible visits with a diagnosed pharyngotonsillitis, a RADT was used in 16,477 (68%) cases and a CRP test was used in 8687 (36%) cases (Figure 2). Most CRP tests (7740; 89%) were used in combination with a RADT, corresponding to 47% of all RADTs. Of these 7740 CRP tests, 7084 (92%) were recorded with the same time signature as the RADT. CRP testing was more common in patients with a negative RADT (3127/5550; 56%) than in patients with a positive RADT (4613/10,927; 42%) (*p* < .001).

In comparison, the total use of these POC tests in the registry during the study period, including visits without diagnosed pharyngotonsillitis, amounted to 36,214 RADTs and 76,015 CRP tests. Of patients tested with a RADT, 20,204/36,214 (56%) were also tested with CRP the same day. CRP testing was more common in patients with a negative RADT (14,117/21,927; 64%) than in patients with a positive RADT (6087/14,287; 43%) (*p* < .001).

### Outcomes of point-of-care tests

In the 24,237 eligible visits with a diagnosed pharyngotonsillitis, 10,927/16,477 (66%) RADTs were positive, and the 8687 CRP tests had a median value of 26 mg/l (IQR 5–65). In patients with a positive RADT, the median CRP was 23 mg/l (IQR 5–59), and in patients with a negative RADT, the median CRP was 31 mg/l (IQR 5–72) (*p* < .001). In the 656 visits where the CRP test and RADT were not recorded with the same time signature, the median CRP was 33 mg/l (IQR 11–71) in the 250 patients with a positive RADT and 49 mg/l (IQR 13–94) in the 406 patients with a negative RADT.

In comparison, an analysis of all POC tests in the registry during the study period, regardless of diagnosis code, showed that 14,287/36,214 (39%) RADTs were positive, and that the 76,015 CRP tests had a median level of 6 mg/l (IQR 0–25). In patients with a positive RADT, the median CRP was 23 mg/l (IQR 5–58); in patients with a negative RADT, the median CRP was 9 mg/l (IQR 0–35).

### Antibiotic prescribing

An antibiotic was prescribed in 18,467/24,237 (76%) of the eligible visits. Nearly all patients with a positive RADT (10,410/10,927; 95%) were prescribed antibiotics, compared with 2407/5550 (43%) patients with a negative RADT (*p* < .001). Of the 6813 patients who underwent neither a CRP test nor a RADT, 5100 (75%) were prescribed an antibiotic.

**Figure 3.**
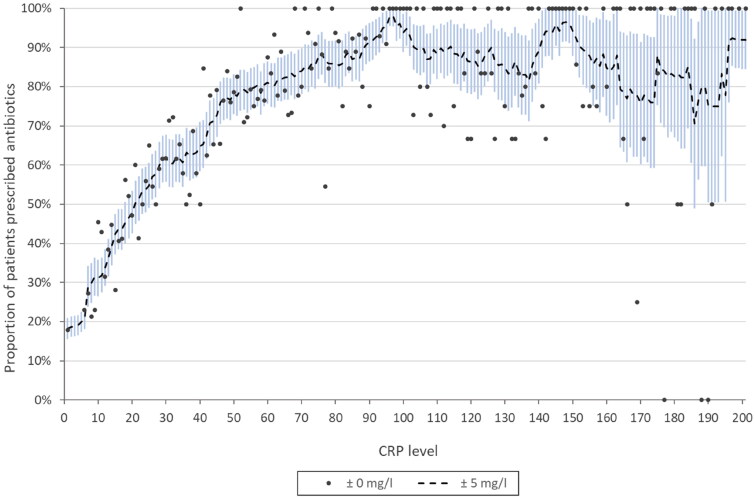
Association between C-reactive protein (CRP) level and antibiotic prescribing in 3127 patients diagnosed with pharyngotonsillitis and with a negative RADT for GAS. The dots represent single CRP levels (± 0 mg/l). The dashed line represents the moving average of each CRP level and the surrounding ± 5 levels, together with a 95% confidence interval for the moving average.

In the 3127 patients with a negative RADT who were also tested with CRP, a logistic regression was performed to study the association between CRP level and prescribing. When adjusted for age and gender, the aOR was 1.032 (95% CI 1.029–1.035; *p* < .001), which can be considered to be a large effect size as this aOR refers to each whole number increase in CRP (range 0–200). The model explained 34% (Nagelkerke R^2^) of the variance in antibiotic prescribing and correctly classified 76% of cases. Half of these 3127 patients were prescribed an antibiotic already at 20 mg/l (Figure 3). Overall, in the 5550 patients with a negative RADT, CRP testing was associated with more than twice the antibiotic prescribing (1768/3127; 57%) compared to no CRP testing (639/2423; 26%) (*p* < .001).

When the regression model above was applied to the 947 patients who underwent CRP testing without a concurrent RADT, CRP level was also associated with prescribing (aOR 1.038; 95% CI 1.032–1.044; *p* < .001). In contrast, when the model was applied to patients with a positive RADT, there was no association between CRP level and prescribing (aOR 1.001; 95% CI 0.998–1.004; *p* = .6).

### Gender, age and temporal trends

Among the 24,237 eligible visits, women accounted for a slight majority (Table 1). However, there were no gender differences in the proportion of RADTs performed, the proportion of positive RADTs, or the proportion of patients prescribed an antibiotic. CRP testing was slightly more common in men than in women.

**Table 1.** Age and gender in relation to point-of-care testing and antibiotic prescribing, 2012–2016.

Group	Number of visits n (column %)	CRP test performed n (%)	RADT performed n (%)	Positive RADT n (%)	Antibiotic prescribing n (%)
Age, years					
<15	9025 (37)	3085/9025 (34)	6386/9025 (71)	5060/6386 (79)	7415/9025 (82)
15–29	6999 (29)	2499/6999 (36)	4570/6999 (65)	2208/2362 (48)	4966/6999 (71)
≥30	8213 (34)	3103/8213 (38)	5521/8213 (67)	3659/1862 (66)	6086/8213 (74)
*p*	<.001^a^	<.001^b^	<.001^b^	<.001^b^	<.001^b^
Gender					
Female	13,626 (56)	4781/13,626 (35)	9242/13,626 (68)	6085/9242 (66)	10,367/13,626 (76)
Male	10,611 (44)	3906/10,611 (37)	7235/10,611 (68)	4842/7235 (67)	8100/10,611 (76)
*p*	<.001^a^	.006^b^	.6^b^	.1^b^	.6^b^
All visits	24,237 (100)	8687/24,237 (36)	16,477/24,237 (68)	10,927/16,477 (66)	18,467/24,237 (76)

*Notes:* CRP: C-reactive protein; GAS: group A streptococci; RADT: rapid antigen detection for GAS. A temporal analysis showed that RADTs and CRP tests were used slightly more often in the first year (2102) of the study (Table 2). However, the proportion of patients who had both a RADT and a CRP test was consistent across years. The proportion of RADTs that were positive differed slightly between the years as did the proportion of patients with a positive RADT that were tested with CRP and patients with a negative RADT that were tested with CRP.

^a^ *p* Values are for one sample Chi square goodness-of-fit tests, comparing samples within age groups and gender groups.

^b^ *p* Values are for Pearson Chi square tests, comparing proportions within age groups and gender groups.

**Table 2.** Annual use of RADTs for GAS and CRP tests in patients diagnosed with pharyngotonsillitis, 2012–2016.

	Year						
	2012	2013	2014	2015	2016	*p*	Total
CRP test performed, n (row %)	2189 (25)	1799 (21)	1468 (17)	1452 (17)	1779 (20)	<.001^a^	8687 (100)
RADT performed, n (row %)	4179 (25)	3567 (22)	2729 (17)	2600 (16)	3402 (21)	<.001^a^	16,477 (100)
Positive RADTs/all RADTs, n (%)	2951 (71)	2412 (68)	1553 (57)	1522 (59)	2489 (73)	<.001^b^	10,927 (66)
Patients with RADT + CRP test, n (%)	2007 (48)	1618 (45)	1265 (46)	1267 (49)	1583 (47)	.050^b^	7740 (47)
Patients with a positive RADT tested with CRP, n (%)	1263 (43)	984 (41)	616 (40)	653 (43)	1097 (44)	.04^b^	4613 (42)
Patients with a negative RADT tested with CRP, n (%)	744 (61)	634 (55)	649 (55)	614 (57)	486 (53)	.006^b^	3127 (56)

*Note:* CRP: C-reactive protein; GAS: group A streptococci; RADT: rapid antigen detection for GAS.

^a^ *p* Values are for one sample Chi square goodness-of-fit tests, comparing samples between years.

^b^ *p* Values are for Pearson Chi square tests, comparing proportions between years.

The median age of the patients in the 24,237 visits was 20 years (IQR 9–36). Children < 15 years old were slightly more often tested with a RADT than were older participants, had a much higher proportion of positive RADTs and were slightly more often prescribed antibiotics (Table 1). However, a multiple logistic regression model, adjusting for gender and RADT outcome, showed no association between age and prescribing (OR 0.999; 95% CI 0.997–1.002; *p* = .7).

## Discussion

### Main findings

In this registry-based study of patients diagnosed with pharyngotonsillitis in PHC, we observed widespread use of both RADTs for GAS (68%) and POC CRP tests (36%). Notably, CRP tests were administered to approximately half of the patients who underwent a RADT, and this practice was common even when the RADT yielded a positive result. Among patients with a negative RADT, we observed a clear association between CRP level and antibiotic prescribing, and CRP testing was associated with a twofold increase in antibiotic prescribing compared to cases where CRP tests were not used.

### Meaning of the main findings

The use of RADTs (68%) was higher than expected. According to the national guideline, testing is recommended only for patients with a Centor score of 3–4 and a presumed benefit of antibiotics, and previous studies on acute sore throat in PHC found that this subgroup constituted 39–44% [5,6,8,25]. The proportion of positive RADTs (66%) was also higher than expected from the national guideline and corresponded to 45% of all patients diagnosed with pharyngotonsillitis. This finding contrasts with meta-analyses, which reported a pooled prevalence of GAS among patients self-presenting to health care providers with acute pharyngotonsillitis to be 14% in adults and 37% in children [26,27]. A GAS prevalence of ≥ 45% lies closer to the previously reported prevalence of 43–48% in patients with a Centor score of 3–4 [5,6,10,28,29]. Two feasible explanations for the high use of RADTs in in this study are that patients with lower scores are also tested, as demonstrated previously [28], and that the choice of diagnosis code may be influenced by the RADT result, meaning patients with a positive result are more likely to be diagnosed with pharyngotonsillitis rather than, for example, upper respiratory infection. Such diagnostic bias could also account for the high proportion of positive tests. Supporting this hypothesis, the eligible visits covered only 45% of all RADTs used in Region Kronoberg during the study period but accounted for 76% of all positive RADTs. However, a more comprehensive data extraction would be necessary to fully explore these numbers, including ICD-10 codes for other respiratory infections and a medical file review. A widespread testing of patients with lower Centor scores than 3–4 will contribute to over-prescribing, as patients with little or no proven benefit from antibiotics will be treated. In addition, as the RADT cannot distinguish infection from carriage, there is also a risk that patients with viral infections and concomitant GAS carriage – which is often the case in children – will receive antibiotics.

Contrary to the national guideline, CRP tests were frequently used, usually as an adjunct to a RADT. Surprisingly, CRP testing was common also in patients with a positive RADT, which seems redundant as almost all patients with a positive RADT were prescribed antibiotics regardless of CRP testing. It is likely that the two tests were ordered at the same time. In patients with a negative RADT, the CRP level appeared to influence the decision to prescribe antibiotics, with half of the patients receiving antibiotics already at astonishingly low CRP levels. A similar association was observed in patients not receiving RADT. The association between CRP level and antibiotic prescribing closely mirrors previous findings in patients with respiratory infections in Danish PHC [14] and aligns with results from a small prospective study in Swedish PHC [20]. It suggests that CRP tests are used in a similar manner in patients with pharyngotonsillitis as in patients with other respiratory infections. Qualitative studies have shown that CRP tests are employed to distinguish between viruses and bacteria, assess the severity of the infection, guide antibiotic prescribing and communicate this assessment with the patient [30]. Moreover, some physicians distrust RADT results, and some consider bacteria other than GAS as important to treat [31]. Although many physicians state that they lack exact CRP cut-off levels for prescribing, CRP is often considered a reliable numerical measure of bacterial infection [31].

The CRP level was higher in patients with a negative RADT than in those with a positive RADT, a contrast to previous studies where patients with GAS exhibited similar or higher CRP levels than those without GAS [17,19,32]. While the study design precludes definitive conclusions about this difference, a possible explanation could be that the decision to use a CRP test in patients with a negative RADT was guided by clinical severity, leading to the selection of patients with more severe symptoms. Moreover, the even greater differences in CRP levels in patients where the two tests were registered with different time signatures suggest a sequential use. Nevertheless, the CRP level was normal in about 25% of the tested patients with a positive RADT and <23 mg/l in approximately 50%, emphasising the inadequacy of CRP as an aetiological test for GAS.

The overall antibiotic prescribing in this study far exceeded expectations outlined in the national guideline and extended well beyond patients with a positive RADT. As highlighted earlier, CRP testing was associated with increased antibiotic prescribing in patients with a negative RADT. However, prescribing rates were also notably elevated in the sizeable cohort of patients who underwent no testing at all. The reason for this latter group cannot be elucidated by this study design, but it seems implausible that all these patients were so severely ill that physicians completely disregarded standard procedures. Instead, a reasonable assumption is that physicians had a high level of confidence in their ability to identify patients requiring antibiotics based solely on clinical presentation.

We believe that the results from this study are valid also outside Region Kronoberg, as PHC is organised in the same way throughout Sweden, with a good access to POC tests. The results from Denmark [14,16] suggest that the situation may also be similar in other countries where POC tests are readily available. However, it is important to note that Swedish physicians do not benefit financially from ordering these tests, which is otherwise a factor than can influence the use. The association between CRP level and antibiotic prescribing is likely to exist elsewhere, although the actual CRP level to which doctors respond may differ depending on various background factors.

### Strengths and limitations

A strength of this study is that we had almost complete data from five consecutive years for the whole population in Region Kronoberg due to the comprehensive electronic medical file system. It is possible that some patients attended other clinics outside Region Kronoberg, causing random missing data, but this is a minor concern as the study focused on the management of patients at the visit rather than following patients over time. Although online doctor consultation with a private eHealth provider is a growing business in Sweden, it was still a largely unknown phenomenon during the study period (2012–2016). Moreover, the study preceded the COVID-19 pandemic and therefore was not affected by altered consultation patterns and behaviours due to the pandemic. A limi­tation with the study is that it focuses on diagnosed pharyngotonsillitis rather than acute sore throat as a symptom. This is a necessity in a registry study based on routine health data, as individual symptoms are not coded as data. Moreover, we were more interested in the management of patients with this defined diagnosis than in patients diagnosed with unspecified upper respiratory infection or ‘pain in throat’, as the national guideline clearly deals with pharyngotonsillitis. Similarly, although some patients may have been misclassified, our interest lay in the management of those patients who received the diagnosis, rather than those who did not. The clinical practice regarding pharyngotonsillitis did not change during the study period, nor did the national guidelines or coding strategies. In Region Kronoberg, there is no reimbursement coupled to diagnosis codes.

This study would have benefitted from clinical information, including the Centor score, as such data provides better understanding of physician behaviour. Unfortunately, such information is coded as raw text in the medical files and was thus impossible to extract from the registry. Physician behaviour can vary greatly on an individual level, and this was another source of bias that we could not control for given this specific dataset. Given how POC tests are supposed to be used according to national guidelines, we can assume that their use was not always random, but probably based on the clinical presentation, i.e. – confounding by indication. It is therefore possible that the CRP value acts as a surrogate marker for disease severity, and that prescribing was based more on the latter than on a certain test result. However, this is contradicted by the fact that half the patients were prescribed an antibiotic already at 20 mg/l, which is a level that should not raise major concerns for the patient’s health. The fact that 42% of the patients with a positive RADT also had a CRP test – which is redundant since a positive RADT is almost synonymous with prescribing – suggests that these tests were ordered simultaneously in advance and not based on clinical presentation. Ideally, there would be a controllable variable measuring the severity of the disease; unfortunately, there is no such variable except the physician’s judgement, if he or she had been asked.

Another limitation with the study is the lack of background data on prognostic factors, such as smoking status, medications, co-morbidity, immunosuppression, a history of relapsing throat infections and patients’ expectations on testing and treatment. These factors are all potential sources of residual confounding, which calls for a more cautious interpretation of the results. For example, physicians might be more prone to prescribe antibiotics at low CRP levels to a patient with immunosuppression or a history of frequent infections. However, from clinical experience, the vast majority of patients attending for a sore throat are young, healthy and not taking any critical medications, and these factors are therefore unlikely to have a significant impact on the outcome. Moreover, the goal with the analysis was not to construct the most accurate model possible to predict antibiotic prescribing at a certain CRP level, but to reveal a larger behavioural pattern.

## Conclusions

The use of RADTs was higher than expected, suggesting both inappropriate use and diagnostic bias. CRP tests were commonly used, even in patients with positive RADTs, and were associated with increased antibiotic prescribing in patients with a negative RADT. The study provides no support that CRP testing, used in addition to RADTs, reduces antibiotic prescribing in pharyngotonsillitis. The study also highlighted the inadequacy of CRP as an aetiological test for GAS, given the normal or low CRP levels in a significant percentage of patients with positive RADTs.
